# Late-stage presentation with decompensated cirrhosis is alarmingly common but successful etiologic therapy allows for favorable clinical outcomes

**DOI:** 10.1371/journal.pone.0290352

**Published:** 2023-08-24

**Authors:** Michael Schwarz, Caroline Schwarz, Lukas Burghart, Nikolaus Pfisterer, David Bauer, Wolfgang Hübl, Mattias Mandorfer, Michael Gschwantler, Thomas Reiberger

**Affiliations:** 1 Department of Internal Medicine IV, Department for Gastroenterology and Hepatology, Klinik Ottakring, Vienna, Austria; 2 Department of Medicine III, Division of Gastroenterology and Hepatology, Medical University of Vienna, Vienna, Austria; 3 Department for Gastroenterology and Hepatology, Department of Internal Medicine IV, Klinik Landstraße, Vienna, Austria; 4 Klinik Ottakring, Institute for Laboratory Medicine, Vienna, Austria; 5 Sigmund Freud University, Vienna, Austria; Al-Azhar University, EGYPT

## Abstract

**Introduction:**

Liver cirrhosis accounts for considerable morbidity and mortality worldwide and late presentation limits therapeutic options. We aimed to assess characteristics of patients with liver cirrhosis at the time of first presentation and during their clinical course.

**Methods:**

Patients with cirrhosis as evident by presence of varices at endoscopy, liver stiffness ≥15kPa at elastography, or ascites requiring paracentesis between Q1/2015-Q2/2020 were retrospectively included. Clinical, laboratory, and imaging data were collected from medical records at presentation and last follow-up.

**Results:**

476 patients were included (alcohol-related liver disease, ALD: 211, 44.3%; viral hepatitis: 163, 34.2%). Of these, 106 patients (22.3%) and 160 patients (33.6%) presented already with Child-Pugh C and MELD >15, respectively, and decompensation events were registered in 50% (238 patients) at baseline, and even in 75.4% of ALD patients.

During a median follow-up of 11.0 (IQR 4–24) months, 116 patients died. Two-year survival was worse for patients with ALD than for viral hepatitis (71.1% vs. 90.2%, log rank p<0.001). We observed the highest percentage of portal-vein thrombosis (30.0%), hepatocellular carcinoma (15.0%), and death (45.0%) in the MAFLD group (n = 20). Patients cured from hepatitis C showed significant improvements in platelet count (147 to 169 G/L, p<0.001) and liver stiffness (26.2 to 17.7 kPa, p<0.001), while ALD patients improved in Child-Pugh score (8.6 to 7.6, p<0.001) during follow-up. With increasing Child Pugh score and MELD, we found increasing serum concentrations of CRP (p<0.001) and an inverse correlation with serum HDL (Spearman’s ρ = -0.573 and -0.529, respectively, p<0.001).

**Conclusion:**

Half of the patients with cirrhosis had decompensated cirrhosis at presentation. This calls for increased awareness and strategies for earlier diagnosis of chronic liver disease and cirrhosis.

## Introduction

Worldwide, liver cirrhosis accounts for substantial mortality and morbidity with approximately two million deaths each year [1,2]. Most common causes are viral hepatitis (i.e. chronic hepatitis B, C or D [CHB, CHC, CHD]), alcoholic liver disease (ALD), and metabolic dysfunction-associated fatty liver disease (MAFLD) [3,4]. Cirrhosis is caused by prolonged damage to the liver parenchyma, subsequent inflammation, and scarring, as well as changes in vascular resistance and perfusion [5,6]. Fibrotic rearrangement of the liver, intrahepatic vasoconstriction, and extrahepatic (splanchnic) vasodilation result in portal hypertension, which causes the formation of venous collaterals (e.g., gastroesophageal varices) and decompensation events like ascites, variceal bleeding, and hepatic encephalopathy [7–9]. Thus, cirrhosis be divided into a compensated and decompensated state, where the occurrence of decompensation is associated with a marked reduction in prognosis with a median survival of about two years after the first event [10,11]. Although there is no predictable sequence of events, ascites is the most common and regularly the first decompensation event [10,12,13].

Despite the severe morbidity of chronic liver disease, its progression to cirrhosis, and the burden on healthcare systems worldwide, underdiagnosis is prevalent [14,15], even though etiological treatment for viral hepatitis or pathogen abstinence for ALD can potentially reverse the damage done [16–19]. Consequentially, patients diagnosed with liver disease for the first time oftentimes already have advanced disease [20,21], and while recent advances in pharmacological therapy for viral hepatitis offer ways to control or cure infections, the global prevalence of cirrhosis due to ALD and MAFLD is rising [4,22–25].

With our paper, we aim to describe the clinical presentation of patients with liver cirrhosis in a real-life setting at a large patient-centered tertiary care center in Vienna, to improve clinical awareness, and, thus, improve patients’ clinical management and prognosis.

## Methods

### Patient cohort

We retrospectively included patients with liver cirrhosis presenting at our tertiary care, non-transplant hospital in Vienna from January 2015 to March 2020. Cirrhosis was defined as any of the following criteria: (i) transient elastography showing liver stiffness ≥15kPa, (ii) presence of gastroesophageal varices, or (iii) presence of ascites. These parameters were chosen to include various stages of compensated and decompensated cirrhosis (significant fibrosis, compensated with varices, decompensated). For (i) vibration-controlled transient elastography, all reports were obtained from our Echosens Fibroscan 502 Touch. The cut-off of 15kPa was chosen in concordance with Baveno 7 guidelines to rule in advanced chronic liver disease with a high likelihood using a non-invasive test [9]. For (ii) the presence of gastroesophageal varices, indicative of portal hypertension, all endoscopy reports from the given timeframe were searched for the keyword “Varize/n” (i.e. “varice/s” in German) using the endoscopy software (ViewPoint, Version 5.6.27.232, GE Health). Each report was reviewed for plausibility and wrong readouts. For (iii) the presence of ascites, as a marker of decompensated liver disease, all laboratory reports on ascites fluid performed in the observed timeframe were extracted from the hospital electronic records database (impuls.kis, version 4.0.3, systema) and non-portal hypertension-related reasons for ascites, like malignant ascites, were excluded. All patients from the three databases meeting at least one of the three inclusion criteria were considered. The chronologically earliest result was chosen as the time of diagnosis (baseline).

Subsequently, the following exclusion criteria were applied: (E1) < 18 years of age, (E2) significant cardiovascular disease, (E3) non-cirrhotic portal hypertension, (E4) extrahepatic malignancy with hepatic metastasis, (E5) insufficient medical records to verify a diagnosis of cirrhosis, (E6) prior diagnosis of cirrhosis outside of the observed timeframe.

### Patient parameters

For the defined baseline date information was retrieved from the electronic medical records. For baseline characteristics, we included laboratory, endoscopy, and imaging reports as well as virus serology and polymerase chain reaction (PCR) results closest to the date of diagnosis (+/- 90 days for laboratory results, +/- 365 for serology and imaging reports, +/- 90 days for HCC/PVT at baseline). The last visit to our institution and/or last laboratory report within the defined timeframe (01/2015-03/2020) was defined as the “last follow-up” endpoint. Etiology of liver cirrhosis, clinical data (sex, weight, height, presence of ascites or hepatic encephalopathy [HE], medication, diabetes mellitus), endoscopy and imaging reports were assessed at baseline.

Data regarding ascites was recorded according to clinical description in medical records, need for diuretics, imaging results, or ascites laboratory work. Spontaneous bacterial peritonitis was defined as a polymorphonuclear neutrophil count (PMN) >250x10^6^ cells/L [26].

The presence of varices was taken from endoscopy reports. Variceal bleeding was defined as either visible bleeding from varices or signs of recent bleeding in the presence of varices and absence of other potential sources of bleeding, especially with bleeding stigmata present on varices (red spots, white nipple sign, etc.). Hepatic encephalopathy (HE) was either taken from medical records at baseline (grade I to IV according to West-Haven Criteria [27]), or defined as the need for HE medication or need for hospitalization due to HE at baseline. Occurrence of portal vein thrombosis (PVT) or hepatocellular carcinoma (HCC) was taken from medical records and imaging reports and assessed from baseline (+/- 90 days) until the latest follow-up. When available, transient elastography results from baseline and follow-up were included. Child Pugh Score and Model for End-Stage Liver Disease (MELD) were calculated from parameters obtained at baseline and latest follow-up. The 2016 United Network for Organ Sharing (UNOS)-MELD was used, which includes sodium in its calculation. Scores were calculated at baseline and at the latest follow-up [28–31].

Baseline imaging reports from ultrasound evaluation, magnetic resonance imaging, and computed tomography at the time of diagnosis were included. Splenomegaly, the presence of ascites or liver nodules as well as portal vein thrombosis were assessed. The report closest to the designated time of diagnosis of cirrhosis (see “Patient cohort”) was included with a maximum range of 365 days from the date. In absence of proper imaging reports within that timeframe, no data was included for baseline. Imaging reports were analyzed until the time of the latest follow-up for the occurrence of PVT or HCC.

Etiologies of cirrhosis were divided into ALD, viral, MAFLD, autoimmune & cholestatic liver disease (autoimmune hepatitis, primary biliary cholangitis, primary sclerosing cholangitis), and “other”. “ALD” was defined as harmful alcohol consumption of more than two standard drinks (e.g., 300ml of beer) or 24g pure alcohol per day as evaluated and mentioned in the medical record [32,33]. The entity of “viral” liver disease was defined as chronic liver disease in the presence of chronic viral hepatitis according to laboratory evaluation by serology and/or PCR at baseline (+/- 365 days) or persistent chronic liver disease after viral clearance. “MAFLD” was defined as body-mass-index (BMI) >30, diagnosis of diabetes mellitus type 2, or steatohepatitis in the biopsy in absence of other causes of cirrhosis. “Other” etiologies included schistosomiasis, drug-induced liver injury (DILI), hemochromatosis, and cryptogenic liver disease.

### Statistics

Variables are presented in absolutes (and percent), in mean (and standard deviation), or in median (and interquartile range). For statistical analysis, Mann-Whitney-U test or Kruskal-Wallis test was performed for the comparison of non-normally distributed independent samples, Wilcoxon sign rank test was used for dependent samples. For correlations, Spearman’s rank correlation coefficient was used for non-normally distributed continuous variables. For survival models, Kaplan-Meier estimates (with log rank test) were used. Data management was performed in Microsoft Excel (Office 2019), statistical analysis was conducted using Excel, IBM SPSS Statistics Version 28, and graphs and figures were designed in GraphPad Prism (version 9.4.1).

### Ethical considerations

The study was approved by the Ethics Committee of the City of Vienna (EK-20-197-VK) and was conducted according to the principles of Good Scientific Practice and the ethical guidelines of the 1975 Declaration of Helsinki (6th revision, 2008). Due to the retrospective character of the study, the Ethics Committee of the City of Vienna waived the requirement for written informed consent.

## Results

### Baseline characteristics

In total, 476 patients were included in the final analysis. Sources of data for the diagnosis of cirrhosis were endoscopy reports (259 patients, 54.4%), transient elastography results (189 patients, 39.6%), and ascites analyses (28 patients, 5.9%). The median age was 56 years (IQR 45.5–67). Our cohort was predominately male (345 patients [72.5%], female 131 [27.5%]). The median body mass index (BMI) overall was 25.7 (IQR 22.7–29.1) and 76 patients (16.0%) had diabetes mellitus at baseline. Patients with MAFLD had the highest median BMI (31.0, IQR 27.1–39.4) and diabetes ratio (15 pts., 75.0%). See Table 1 and S1 Fig in S1 File.

**Table 1:** Baseline characteristics of patients at the time of diagnosis of liver cirrhosis. Our cohort was predominantly male with a median age of 55 years at the time of diagnosis. The most common etiologies of cirrhosis were alcoholic liver disease (ALD) and viral hepatitis and the baseline characteristics of these subcohorts are compared on the two rightmost columns. Patients with ALD usually presented at later stages of liver disease with higher CPS and MELD. Most deaths occurred in this group (68 patients, 32.2%).

Characteristic	Overall	ALD	viral
Number of patients	476	211	163
Age median (IQR)	55 years (45–65)	58 (49–68)	50 (42–57)
Sex	345 male (72.5%),	155 male (73.5%)	116 (71.2%)
Diabetes mellitus	76 (16.0%)	31 (14.7%)	20 (12.3%)
BMI median (IQR)	25.7 (IQR 22.7–29.1)	25.2 (21.9–28.5)	25.6 (22.6–28.4)
Child Pugh Score median (IQR)	7 (IQR 5–9)	9 (7–10)	5 (5–6)
MELD median (IQR)	12 points (IQR 9–19)	16 (12–22)	9 (8–11)
Platelet count G/L median (IQR)	124 (80.5–184)	119 (77–170)	141 (88–189)
Follow-up months median (IQR)	11.0 (4–24)	10 (2–25)	14 (6–26)
Death in follow-up (%)	115 (24.2%)	68 (32.2%)	17 (10.4%)
Lost to follow-up (%)	154 (32.4%)	97 (46.0%)	23 (14.1%)
Liver disease etiology • ALD • viral • ALD + viral • MAFLD • autoimmune/cholestatic • MAFLD + viral • others	211 (44.3%) 163 (34.2%) 50 (10.5%) 20 (4.2%) 10 (2.1%) 5 (1.0%) 17 (3.6%)	-	-

### Etiologies

The most common etiologies of cirrhosis were ALD (211 patients, 44.3%), viral hepatitis (163 patients, 34.2%), viral hepatitis and ALD (50 patients, 10.5%), and MAFLD (20 patients, 4.2%). Of the 50 patients (10.5%) who had an overlap between alcoholic and viral liver disease, 43 patients had ALD and CHC, while 7 had ALD and CHB. Five patients (1.1%) had MAFLD and CHC. The largest singular etiologies excluding overlaps were ALD (211 patients, 44.3%) and CHC (147 patients, 30.9%). See Table 1.

In total, 218 patients had cirrhosis (in part or entirely) due to viral hepatitis. This included 142 patients (65.1% of 218) with CHC and 23 patients (7.3%) with CHB. Several patients had overlaps between ALD and CHC (43, 19.7%) or CHB (7, 3%) or NAFLD and CHC (5, 2.3%). Four patients (0.8%) had a coinfection with hepatitis B and D and three patients had CHC and concomitant infections with the human immunodeficiency virus (HIV). Seven patients (1.5%) had cirrhosis after successful hepatitis C eradication at baseline. In total, 179 patients (37.5%) were viremic for hepatitis C virus at baseline defined by serum HCV RNA greater than 15 IU/mL detected by PCR. We further evaluated the therapeutic course of 142 patients with CHC (excluding overlaps with other causes of cirrhosis). Four patients died before treatment could be initiated and 13 patients declined therapy. The remaining 125 patients received treatment with direct-acting antivirals (DAAs) and 109 patients (87.2%) achieved sustained virologic response 12 weeks after completion of antiviral therapy (SVR12). Twelve patients (9.5%) were lost to follow-up and three patients (2.4%) died due to reasons unrelated to treatment before SVR12 could be confirmed. One patient was still on post-treatment surveillance in the observed timeframe. According to modified intention-to-treat (mITT) analysis, excluding patients lost to follow-up and those who died due to unrelated reasons, SVR12 rate was 100% (109/109). Among the 109 patients who achieved SVR12, 19 patients (17.4%) had decompensated cirrhosis, predominantly with ascites (16 of 19 patients). Patients with overlapping etiologies, like concomitant CHC and ALD, also receive treatment with DAAs, but were not included in this analysis. See S3 Table in S1 File.

### Decompensation events, portal vein thrombosis, hepatocellular carcinoma

**Fig 1:**
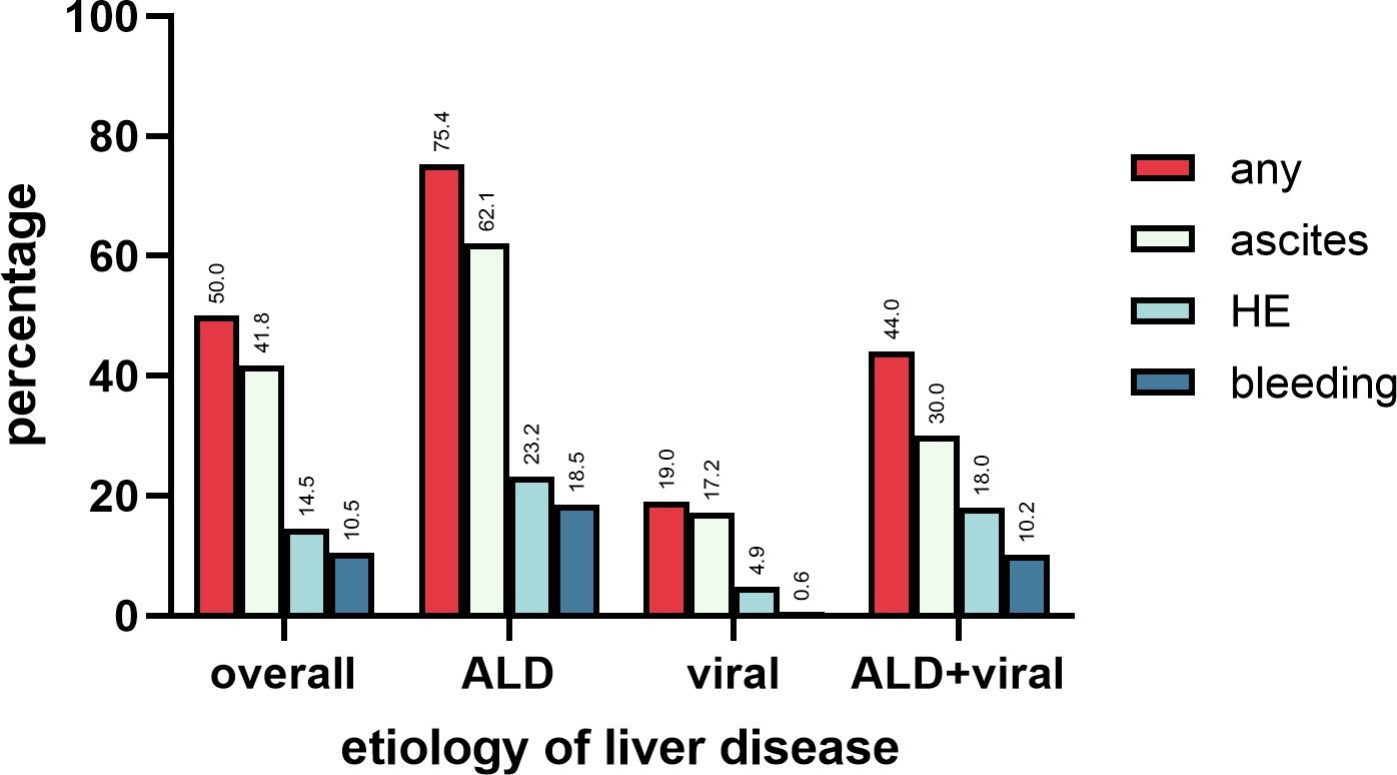
Decompensation events present at the time of diagnosis of liver cirrhosis. In total, half of the patients (50.0%) already had decompensated cirrhosis at baseline. Decompensations were most common in ALD patients (75.4%), while patients with cirrhosis due to viral hepatitis less frequently presented with decompensations at baseline (19.0%). Patients with both ALD and viral hepatitis as causes of cirrhosis showed similar decompensation rates as the overall population.

At the time of diagnosis of cirrhosis, 238 patients (50.0%) already had decompensated advanced chronic liver disease with ascites, variceal bleeding, and/or hepatic encephalopathy. Most common decompensation event at baseline was ascites (198 of 238 patients, 83.2%), followed by hepatic encephalopathy (69 patients, 30.0%), and variceal bleeding (50 patients, 21.0%). Complication ratio at baseline were significantly higher in the ALD than in viral cirrhosis cohort (ALD: 159/211 patients, 75.4% vs. viral cirrhosis: 31/163 patients, 19.0%; p <0.001). See Fig 1 and Table 2.

**Table 2:** Chronic liver disease-related complications and decompensation events present at the time of diagnosis of cirrhosis; overall and according to etiology. “Any complications” were defined as ascites, hepatic encephalopathy (HE) and/or variceal bleeding. Other liver-related events were spontaneous bacterial peritonitis (SBP), hepatocellular carcinoma (HCC) and portal vein thrombosis (PVT). Half of the patients (238, 50.0%) had some decompensation event at baseline, with the most common being ascites (199, 41.8%). Cirrhosis due to ALD had the highest ratio of decompensations at baseline (75.4%), especially most variceal bleeding events–absolute and relative to cohort size (39 bleeding events, 18.5%).

	any	Ascites	variceal bleeding	HE	SBP	HCC	PVT
**overall**	238 (50.0%)	199 (41.8%)	50 (10.5%)	69 (14.5%)	11 (2.3%)	17 (3.6%)	14 (2.9%)
**ALD**	159 (75.4%)	131 (62.1%)	39 (18.5%)	49 (23.2%)	6 (2.8%)	6 (2.8%)	5 (2.4%)
**viral**	31 (19.0%)	28 (17.2%)	1 (0.6%)	8 (4.9%)	4 (2.5%)	3 (1.8%)	3 (1.8%)
**MAFLD**	13 (65.0%)	13 (65.0%)	2 (10.0%)	1 (5.0%)	0 (0.0%)	3 (15.0%)	4 (20.0%)
**AIH/cholestatic**	5 (50.0%)	5 (50.0%)	1 (10.0%)	2 (20.0%)	0 (0.0%)	0 (0.0%)	0 (0.0%)
**mixed**	23 (41.8%)	16 (29.1%)	5 (9.3%)	9 (16.4%)	1 (1.8%)	5 (9.1%)	1 (1.8%)
**other**	7 (40.2%)	6 (35.3%)	2 (11.8%)	0 (0.0%)	0 (0.0%)	0 (0.0%)	1 (5.9%)

Abbreviations: AIH/cholestatic = autoimmune hepatitis or cholestatic liver disease, ALD = alcohol-related liver disease, HCC = hepatocellular carcinoma, HE = hepatic encephalopathy, MAFLD = metabolic dysfunction-associated fatty liver disease, mixed = more than one etiology of liver disease, PVT = portal vein thrombosis, SBP = spontaneous bacterial peritonitis. “Other” includes cryptogenic cirrhosis, hemochromatosis, drug induced liver injury, and schistosomiasis.

A total of 23 patients (4.8%) were diagnosed with HCC: seventeen patients (3.6%) at baseline and 6 patients (1.3%) during FU. Distribution among etiologies was: six patients with ALD, four patients with ALD and viral cirrhosis, three patients with viral cirrhosis, three patients with MAFLD, and one patient with concomitant MAFLD and viral cirrhosis. In total, most HCCs occurred in the ALD cohort (6/211, 2.8%), but relative to cohort HCC ratio was highest in the MAFLD cohort (3/20, 15.0%). No patient was eligible for curative treatment, but 8 patients received locally ablative therapy) and 3 patients received systemic treatment with tyrosine-kinase inhibitors. The other HCC patients received best supportive care.

Presence of PVT was detected in 14 patients (2.9%) at baseline and in seven (1.5%) at FU, amounting to a total of 21 PVTs (4.4%) among our patients. Etiologies were: six of 21 (28.6%) patients with ALD, five (23.8%) patients with viral cirrhosis, two (9.5%) patients with ALD and viral cirrhosis, six (28.6%) patients with MAFLD, one (4.8%) patient with MAFLD and viral cirrhosis, and one (4.8%) with cryptogenic cirrhosis. Relative to cohort size, PVT rate was highest in the MAFLD cohort (6/20, 30.0%). Two patients had cavernous transformation of the portal vein at diagnosis. Six (42.9%) of the 14 PVT diagnosed at baseline and three (42.9%) of the seven PVT diagnosed during follow up occurred together with a HCC.

### Scores and laboratory reports

At diagnosis and across all entities, the median Child Pugh score (CPS) and MELD score were A7 (IQR 5–9) and 12 (IQR 9–19), respectively. At baseline, 106 patients (22.3%) had a CPS of C and 160 (33.6%) a MELD score >15. We found strong inverse correlations for both CPS and MELD with platelet count (in G/L, Spearman’s ρ = -0.168 and -0.266, p <0.001 for both) and hemoglobin concentration (in g/dL, ρ = -0.564 and -0.513, p < 0.001). See Fig 4.

ALD patients presented at later stages of liver disease with a median CPS of B9 (IQR 7–10) and a MELD of 16 (IQR 12–22), as compared to patients with viral cirrhosis (median CPS A5 [IQR 5–7], MELD: 9 [IQR 8–11]). In the ALD cohort, 79 patients (37.4%) had a Child Pugh Stage C and 113 (53.6%) a MELD score >15 at baseline, while for viral cirrhosis this was only the case for 9 (5.5%) and 17 (10.4%) patients, respectively. See Table 3.

**Table 3:** Child Pugh Score (CPS) and Model for End-stage Liver Disease (MELD) Score at the time of diagnosis of cirrhosis and according to etiology of liver disease. At baseline, 22.1% of patients already had a Child Pugh Stage C and 33.4% had a MELD Score greater than 15. Patients with cirrhosis due to ALD usually presented with the highest CPS and MELD scores.

	median CPS, MELD	CPS A	CPS B	CPS C	MELD <10	MELD 10–15	MELD >15
**overall**	A7, 12	205 (43.1%)	166 (34.9%)	105 (22.1%)	154 (32.4%)	148 (31.1%)	159 (33.4%)
**ALD**	B9, 16	37 (17.5%)	95 (45.2%)	79 (37.4%)	27 (12.8%)	68 (32.7%)	113 (53.6%)
**viral**	A5, 9	120 (73.6%)	34 (20.9%)	9 (5.5%)	94 (57.7%)	41 (25.2%)	17 (10.4%)
**MAFLD**	B8, 12	5 (25.0%)	12 (60.0%)	3 (15.0%)	5 (25.0%)	7 (35.0%)	7 (35.0%)
**AIH/cholest.**	B7, 10.5	4 (40.0%)	4 (40.0%)	2 (20.0%)	2 (20.0%)	4 (40.0%)	4 (40.0%)
**mixed**	A6, 10.5	30 (54.5%)	15 (27.3%)	10 (18.2%)	23 (41.8%)	7 (35.0%)	13 (23.6%)
**other**	A6, 14	10 (55.6%)	6 (33.3%)	2 (11.1%)	3 (16.7%)	9 (50.0%)	6 (33.3%)

**Fig 2:**
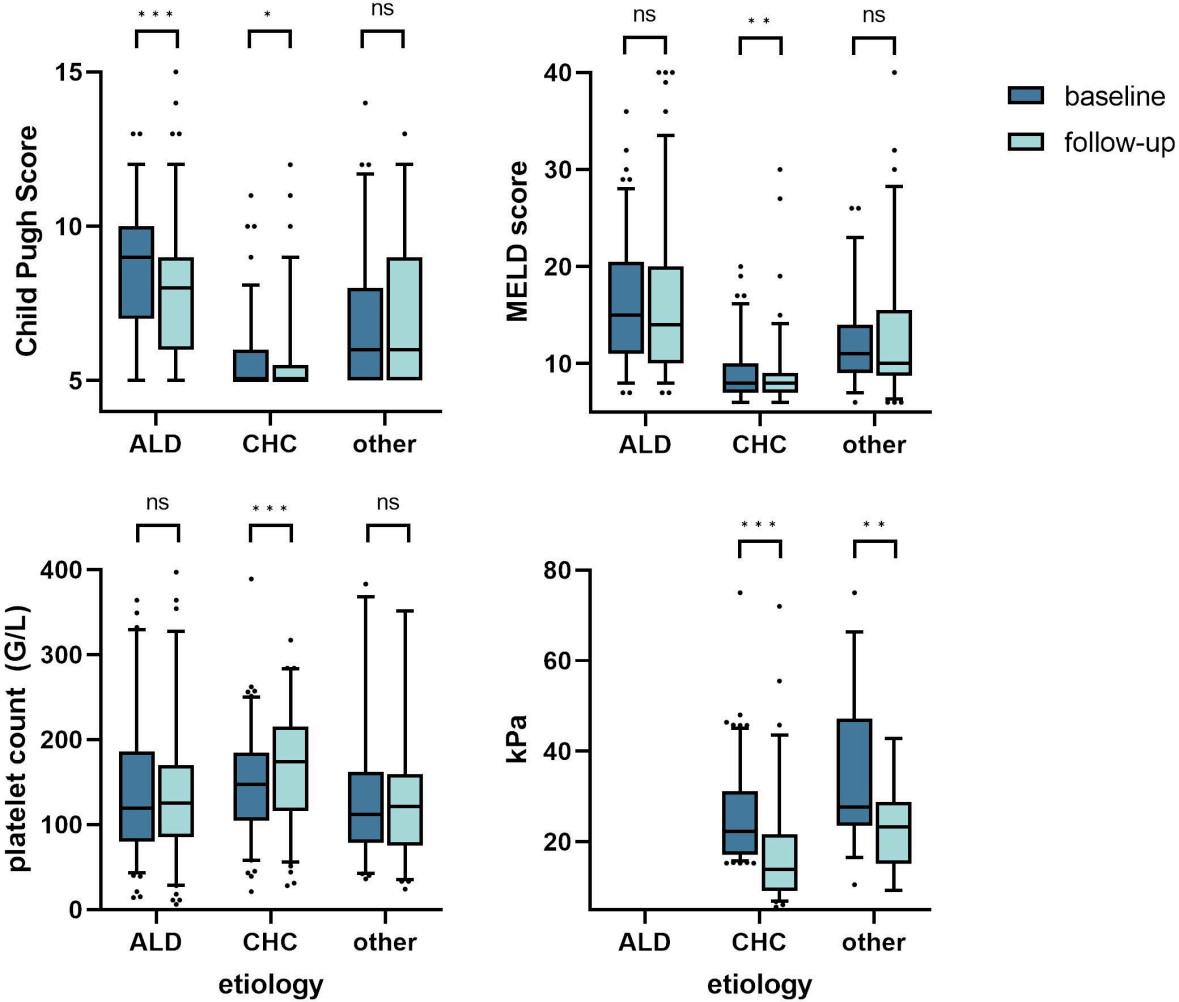
Changes from baseline to last follow-up in Child Pugh Score (CPS), MELD score, platelet count, and liver stiffness (transient elastography in kPa) for patients with cirrhosis due to alcoholic liver disease (ALD), chronic hepatitis C (CHC) or other reasons. Box and whiskers graph (5–95% percentile whiskers) of paired measurements (diagnosis and latest follow-up) for the main singular etiologies of cirrhosis in our cohort: ALD, CHC and “other”. Patients with ALD had higher scores at baseline, but showed improvement in CPS over time. Patients with chronic hepatitis C presented at earlier stages of liver disease. We found a significant effect of hepatitis C virus eradication (SVR12) on CPS and MELD, on platelet count as well as on liver stiffness. For the ALD cohort, not enough paired measurements of liver stiffness were obtained. Level of significance: * = p <0.05, ** = p < 0.01, *** = p < 0.001.

When comparing baseline values to the latest follow-up in paired measurements, CPS significantly decreased in the overall population (CPS: 7.1 vs. 6.8, p = 0.028; MELD: 12.8 vs. 12.9, n.s.; note that these values may differ from baseline as only values with follow-up were included to account for patients lost to follow-up). When looking at singular etiologies, ALD patients also showed a significant improvement in CPS, but not in MELD (CPS: 8.6 vs. 7.8, p < 0.001; MELD: 16.5 vs. 15.8, p = 0.186). CHC patients who achieved SVR12 (n = 109) showed significant reduction in both scores (CPS: 5.8 vs. 5.6, p = 0.035; MELD: 9.1 vs. 8.9, p = 0.008)and significant improvement in platelet count (146.6 vs. 168.6 G/L, p < 0.001) and liver stiffness (26.2 vs. 17.7 kPa, p < 0.001). See Fig 2, and S2 Table in S1 File.

**Fig 3:**
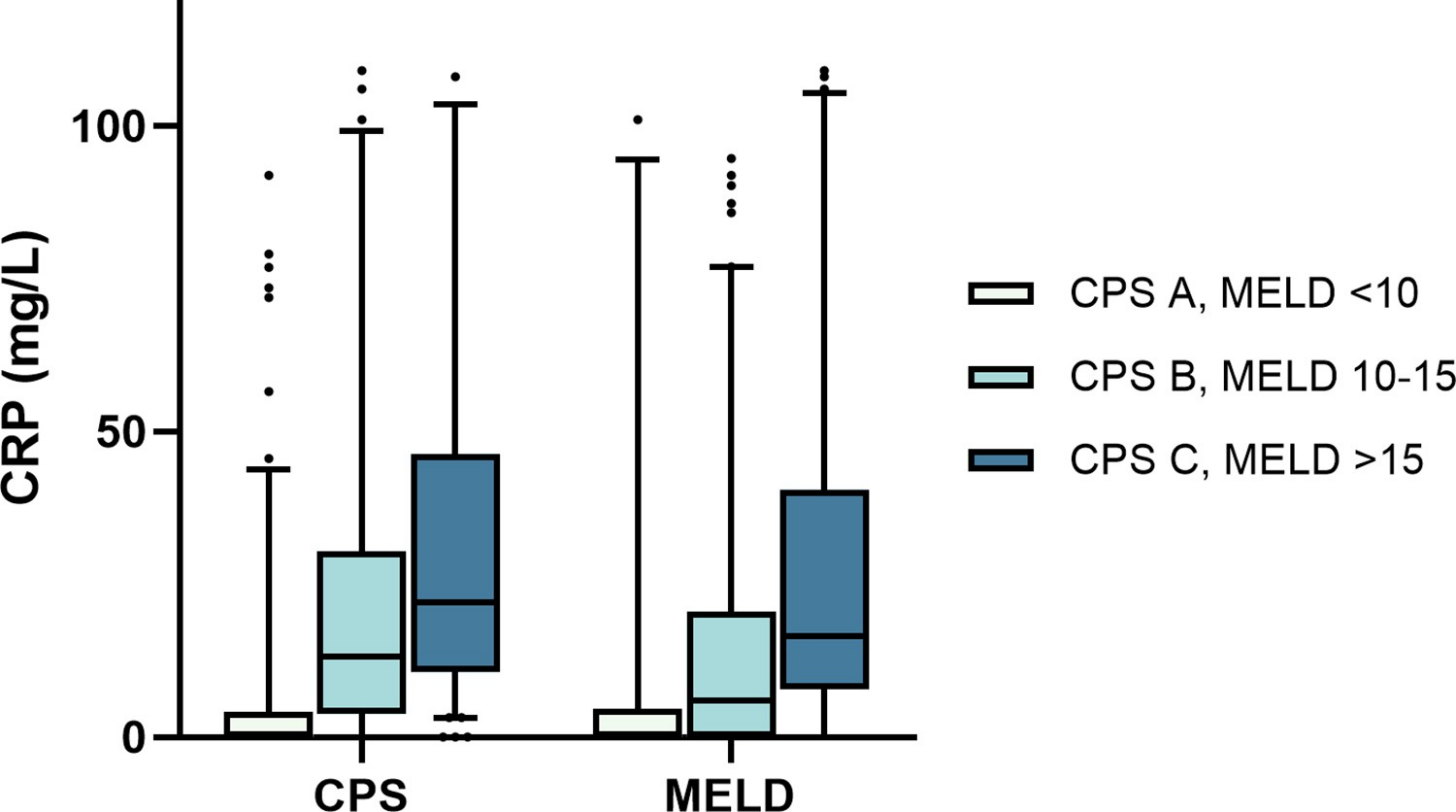
Systemic inflammation increased with liver disease. We found increasing levels of c-reactive protein (CRP, in mg/L) as a marker of systemic inflammation with increasing severity of liver disease, measured in Child-Pugh Score (CPS) and Model for End-stage Liver Disease (MELD) score (Box and whiskers, 5–95% percentile; p < 0.001).

Regarding systemic inflammation measured by serum C-reactive protein (CRP, normal range 0–5.0 mg/L) levels, we found increasing CRP concentrations across both MELD strata (defined as <10, 10–15, and >15) and CPS A/B/C (median CRP for MELD <10: 0.0mg/L [IQR 0.0–4.7], MELD 10–15: 6.1 [IQR 0.0–20.5], MELD >15: 16.5 [IQR 7.9–40.5], p < 0.001; median CRP for CPS A: 0.0 [IQR 0.0–4.3], CPS B: 13.2 [IQR 3.8–30.4], CPS C: 22.0 [10.7–46.4], p < 0.001). See Fig 3. Patients with ALD had significantly higher CRP levels than patients with viral cirrhosis (median 15.8 [IQR 7.2–34.6] vs. 0.0 [0.0–4.4], p < 0.001).

**Fig 4:**
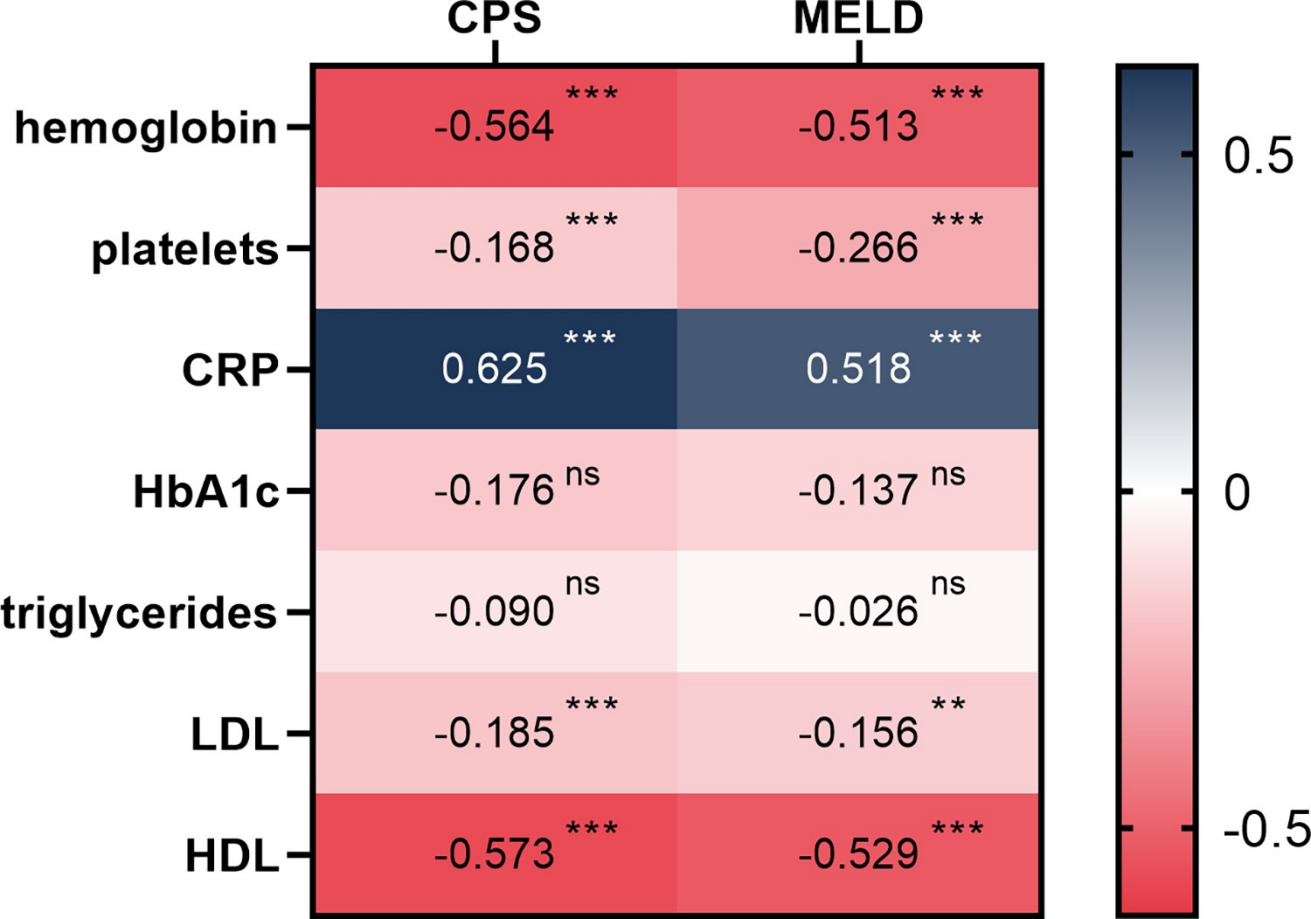
Spearman correlation matrix for CPS and MELD (two-tailed) at the time of diagnosis. When comparing score and laboratory parameters at baseline, we found a strong correlation between CPS and MELD for CRP as a marker for systemic inflammation. We also found a strong inverse correlation for serum HDL levels with disease progression–expressed as increasing CPS and MELD. The association with LDL was not as strong and no significant correlation was found for triglycerides or HbA1c. Patients with advanced chronic liver disease were regularly anemic, as shown here in a strong inverse correlation with hemoglobin levels and CPS/MELD. Level of significance: * = p <0.05, ** = p < 0.01, *** = p < 0.001.

We found a substantial part of patients with cirrhosis had dyslipidemia at the time of diagnosis: 275 patients (57.8%) had reduced high-density lipoprotein (HDL, defined as <40mg/dL), 121 patients (25.4%) had increased low-density lipoprotein (LDL, defined as >100mg/dL) and 133 patients (27.9%) had increased triglyceride levels (defined as >150mg/dL). HDL levels differed greatly amongst etiologies (p < 0.001 overall) with the greatest difference found between patients with ALD or viral cirrhosis (median 27.0 [15.0–41.0] vs. 42.0 [IQR 32.0–53.3], p < 0.001 after Bonferroni correction for multiple testing). HDL levels also decreased significantly with liver disease (p < 0.001 across CPS stages and MELD strata [<10, 10–15, >15]). A similar effect was seen for LDL levels, with significant reduction across CPS (p = 0.002), but not across MELD strata (p = 0.052). LDL was highest in the “cholestatic liver disease and autoimmune hepatitis” subcohort and lowest for ALD (median 96.0 [IQR 46.2–94.7] vs. 96.0 [77.7–144.1], p = 0.02 after correction). See Table 4. We found a strong inverse correlation for HDL serum levels and CPS and MELD score (Spearman’s ρ = -0.573 and -0.529, respectively, p < 0.001). The effect was less intense, but still significant for LDL levels (ρ = -0.185 for CPS, p = <0.001; ρ = -0.156 for MELD, p = 0.001) and not significant for serum triglycerides. See Fig 4.

**Table 4:** Dyslipidemia and liver disease (values in median (IQR)). Shown here are serum levels of LDL, HDL, and triglycerides of the included patients, divided by etiologies, Child Pugh Score (CPS), and MELD strata. We found steep declines in HDL levels with the progression of liver disease. Consequentially, ALD patients, who in general presented at more advanced stages of liver disease, had the lowest HDL levels. We found similar patterns for LDL and triglycerides, but not as significant. LDL levels were highest in the “cholestatic and AIH” subcohort, mainly due to the highly elevated levels among patients with primary biliary cholangitis.

	LDL	p	HDL	p	triglycerides	p
**ALD**	62.8 (46.2–94.7)	**0.009**	27.0 (15.0–41.0)	**<0.001**	99.5 (76.0–144.8)	0.141
**viral**	70.8 (55.0–98.8)		42.0 (32.0–53.3)		107.5 (77.5–157.5)	
**MAFLD**	69.1 (47.4–109.3)		30.5 (17.8–40.0)		107.5 (79.8–150.5)	
**cholestatic and AIH**	96.0 (77.7–144.1)		39.5 (22.8–55.5)		107.0 (75.3–141.5)	
**Other**	75.4 (34.2–97.9)		43.5 (22.8–55.5)		150.0 (101.5–200.5)	
**CPS A**	72.0 (56.0–99.1)	**0.002**	45.0 (35.0–56.8)	**<0.001**	118.0 (81.0–164.0)	0.060
**CPS B**	66.3 (42.0–92.8)		30.0 (18.0–41.0)		99.0 (76.0–150.3)	
**CPS C**	58.0 (44.0–83.0)		18.0 (12.0–32.0)		99.0 (76.0–141.0)	
**MELD <10**	71.1 (56.8–98.2)	0.052	45.0 (34.0–58.0)	**<0.001**	125.5 (81.3–165.5)	**0.002**
**MELD 10–15**	67.0 (47.3–93.3)		36.5 (26.5–49.0)		94.5 (75.0–126.5)	
**MELD >15**	60.6 (41.3–95.6)		19.0 (11.0–31.0)		111.0 (78.8–169.0)	

### Endoscopy reports

Endoscopy reports were available for 338 patients at the time of diagnosis. In total, 293 patients had varices at baseline (86.7% of 338). Of these, 284 patients (84.3% of 338) had esophageal varices, 82 of which (24.3%) were described as “large”. Forty-one patients (12.1%) had isolated gastric or gastroesophageal varices and 31 of these patients had no concurrent esophageal varices. Portal hypertensive gastropathy (PHG) was seen in 155 (45.9%) cases. Most patients with ALD had varices already present at the time of diagnosis of cirrhosis (197 of 211, 93.4%). Endoscopy reports in patients with CPS A found PHG in 39 of 89 (43.8%) cases, with CPS B in 62 of 140 (44.3%), and with CPS C in 54 of 100 (54.0%). At baseline, 51 patients (15.1% of 338 endoscopy reports or 10.7% of all patients) presented with variceal bleeding (or suspected variceal) and most of these patients had ALD (39, 76.5% of 51). Other etiologies were: four patients with concomitant ALD and viral cirrhosis (7.8%), two with MAFLD (3.9%), two with viral cirrhosis (3.9%), two with other causes (3.9%), and one patient with cholestatic liver disease (2.0%). A total of 36 patients received variceal band ligation at baseline and 8 received cyanoacrylate glue injection for gastric varices. A second bleeding event occurred in 10 of 51 patients (19.6%). Four patients (7.8% of 51) died during or shortly after high-urgency endoscopy for variceal bleeding: three patients due to uncontrollable bleeding (5.9%) and one patient had a myocardial infarction and pulmonary embolism during cyanoacrylate glue injection (2.0%).

### Imaging reports

A total of 398 baseline imaging reports were analyzed, consisting of 288 sonography reports (72.4%), 93 computed tomography reports (23.4%), and 17 magnetic resonance imaging reports (4.3%). In 33 patients (6.9%) “liver nodules” (benign, suspect, or potentially malignant) were described at baseline and 17 (51.5% of 33 patients) were later confirmed as hepatocellular carcinoma (HCC). Overall, 222 patients (46.5%) had splenomegaly with a median spleen size of 15.0cm (quartile range 13.0–16.0cm).

### Survival

For overall survival analysis, “death” was the endpoint. Over a median follow-up of 11.0 months (interquartile range 4–24) 154 patients (32.4%) were lost to follow-up and 115 (24.2%) died. The two-year overall survival across all etiologies was 77.9% (371 of 476 patients). Eight patients (1.7%) were evaluated for orthologous liver transplantation and one patient received a donor liver during follow-up.

Patients with decompensated liver disease at the time of diagnosis had a worse prognosis than compensated patients (24 months survival: 67.2% vs. 88.7%, log rank p < 0.001). Presence of esophageal or gastric varices as a marker of portal hypertension also impaired prognosis compared to patients without varices (24 months survival: 68.6% vs. 92.9%, log rank p < 0.001). Increased serum levels of CRP, defined as elevated above the median CRP level in our cohort of 6.8mg/L, also had a negative effect on survival (24 months survival: 67.5% vs. 88.3%, log rank p < 0.001). We also found a negative effect of low levels of HDL on prognosis (cut-off set at <40mg/dL; 24 months survival: 72.7% vs. 87.2%, log rank p < 0.001).

**Fig 5:**
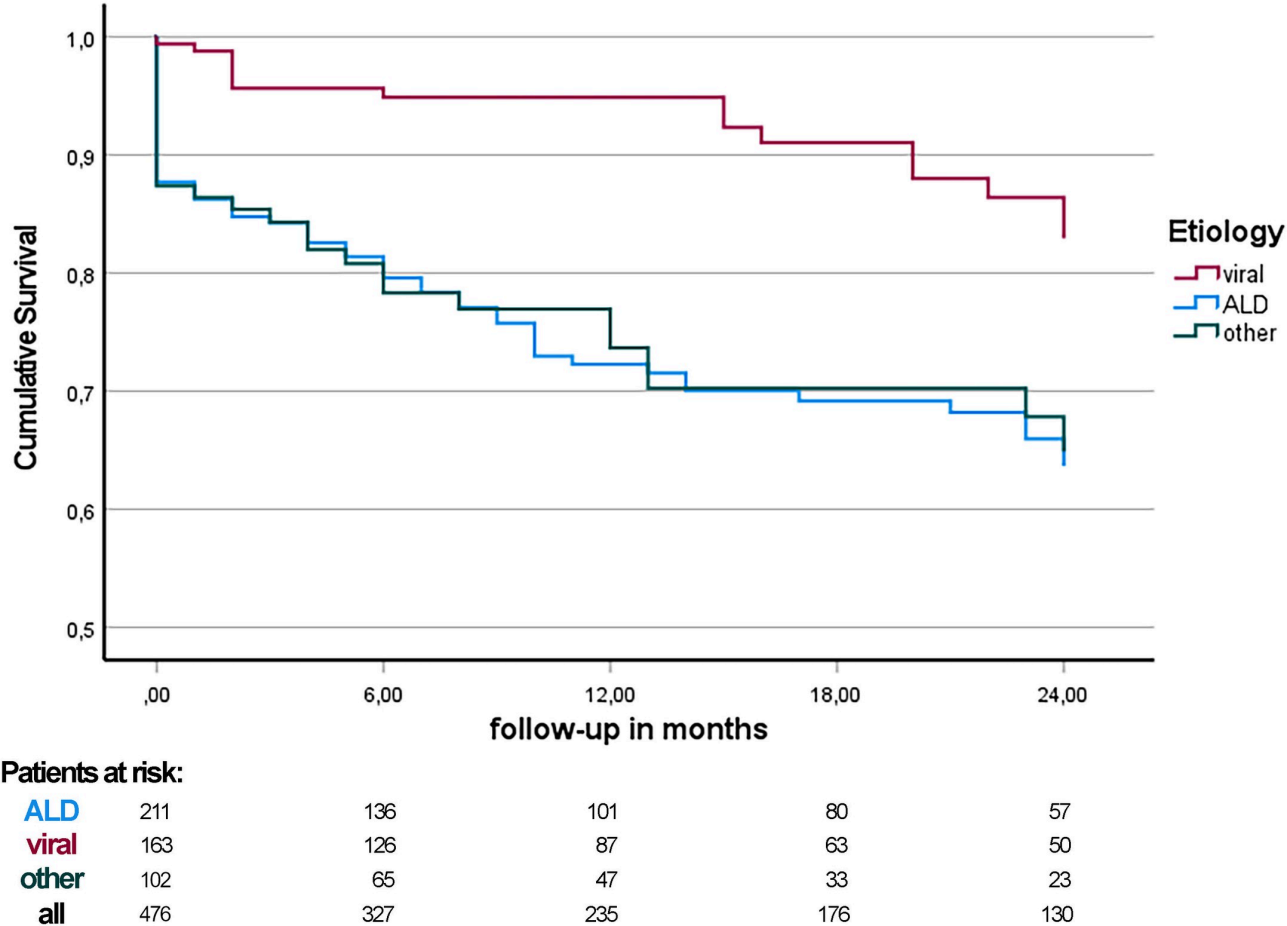
Kaplan-Meier estimates of two-year survival in patients with liver cirrhosis due to alcoholic liver disease and viral hepatitis. After a follow-up of twelve months, the overall survival in the viral hepatitis group was significantly better (95.1% vs. 75.8%, log rank p < 0.001). After 24 months, 90.2% of patients in the viral hepatitis cohort were still alive compared to 71.1% in the ALD cohort (log rank p < 0.001).

Regarding CPS at baseline, two-year survival for CPS A was 92.2% (189 of 205 patients), 74.5% for B (123 of 165 patients) and 55.7% for CPS C (59 of 106 patients; log rank p < 0.001). According to the MELD strata at baseline, two-year survival was 90.3% for MELD <10 (139 of 154 patients), 84.4% for MELD 10–15 (124 of 147 patients), and 59.4% for MELD >15 (95 of 160 patients; log rank p < 0.001). Most deaths occurred in the ALD cohort (68 patients, 58.6% of total deaths). Consequentially, the two-year overall survival was significantly lower in the ALD cohort (71.1%, 150 of 211 patients) compared to the viral cohort (90.2%, 147 of 163 patients, log rank p <0.001). Relative to cohort size, the highest ratio of deaths was observed in the MAFLD cohort (9 of 20, 45.0%). See Fig 5 and S1 Table in S1 File.

## Discussion

Liver cirrhosis marks common endpoint of various entities of chronic liver disease. Even though liver diseases like MAFLD are common and viral hepatitis today is easily controllable or even curable, the diagnosis of liver disease oftentimes comes late and at advanced stages with potentially irreversible liver damage [20,21]. In our cohort of real-world patients with liver cirrhosis, we made an alarming observation: at the time of diagnosis of cirrhosis, half of our patients already had decompensated liver disease, a third of our patients had a MELD score greater than 15, and a quarter had a Child Pugh Stage of C. This dramatically limited the therapeutic options for our patients and only one was eligible for liver transplantation. It is not surprising that decompensated patients have a worse prognosis than patients with compensated liver disease, however, their big share of our overall cohort highlights the necessity and urgency for earlier diagnosis.

Despite being a hepatitis C treatment center, the most common cause of cirrhosis we observed was alcoholic liver disease and hese patients performed significantly worse at baseline when compared to patients with viral cirrhosis. Almost all ALD patients had varices at baseline (93.3%), a high rate of decompensations (75.4%), and consequentially, unfavorable prognosis. These observations are concerning for two reasons: firstly, while viral hepatitis can be treated etiologically–pharmacologically controlled for hepatitis B and even cured for hepatitis C–and consequentially, the global prevalence of viral cirrhosis is decreasing, the number of cases of cirrhosis due to ALD (and MAFLD) are rising [24,25]. Secondly, patients with ALD generally present at later stages and have worse prognosis than patients with non-ALD cirrhosis [34,35], which was also the case in our Viennese cohort. Together, late presentation with limited therapeutic options, frequent readmissions, and increasing prevalence will increase the burden on healthcare systems worldwide [36–38]. However, a bias in our data has to be discussed as the majority of patients with viral hepatitis had an elective therapy appointment, while ALD patients mostly presented in non-elective settings and also had the most deaths, thus limiting the follow-up data. Still, both groups greatly benefited from linkage to care as shown by significant decreases in MELD and CPS. If possible, patients received etiological treatment for their liver disease like antiviral agents or were referred to programs/centers specializing on alcohol withdrawal/abstinence (“Anton Proksch Institut”, “Grüner Kreis”, and other local centers). Patients with acute alcohol withdrawal syndrome received symptomatic therapy with benzodiazepines on the ward. Patients were followed regularly at our outpatient clinic, where the importance of lifestyle modifications (weight loss and regular activity), alcohol abstinence and/or syringe exchange programs was addressed. Visits included laboratory analysis and regular diagnostic imaging for HCC surveillance. Patients received treatment with non-selective betablockers (propranolol, carvedilol) in presence of portal hypertension and diuretics (furosemide, torasemid and/or spironolactone, eplerenone) for ascites.

We found that CHC patients who achieved SVR12 showed a significant reduction in liver stiffness, which in turn translates into decreased portal pressure, fewer decompensation events, and improved liver-related survival [16,39–43]. Some of the included patients (7, 1.5%) had already received successful antiviral treatment for chronic hepatitis C before diagnosis of cirrhosis at our center, but still had a transient elastography over 15kPa. Importantly, these patients who achieve SVR12, but retain an increased liver stiffness, may still be at risk for the development of HCC and should remain on surveillance [44,45]. The improved platelet counts we observed after SVR12 might also be a direct effect of viral clearance [46].

Dyslipidemia, defined by either low HDL or high LDL levels or high levels of serum triglycerides, is commonly found in all chronic liver disease [47–50]. In our cohort, we found that low HDL levels were most prominent in the ALD cohort and that low HDL correlated with increased MELD and CPS, and was predictive of a limited prognosis during follow-up, suggesting low HDL as a “red flag” of chronic liver disease [26]. Although we found significant differences in LDL levels across etiologies, the main driver behind this were the elevated levels among patients with primary biliary cholangitis (PBC) in comparison to other etiologies–a well-known effect of the disease [51].

Systemic inflammation is associated, if not a major contributor, to liver cirrhosis and increases with disease progression [6,52,53]. We found increasing CRP levels with both increasing CPS and MELD and elevated CRP levels had a negative effect on prognosis. Furthermore, ALD patients had high CRP levels at baseline, thus highlighting this high-risk population among patients with cirrhosis.

Cirrhosis in MAFLD is oftentimes diagnosed at a higher age and thus, more advanced stage of liver disease than in other etiologies like ALD or viral cirrhosis, limiting the therapeutic options [54]. Furthermore, while MAFLD is the fastest growing cause of HCC in the USA and parts of Europe, patients with MAFLD can develop HCC even in absence of cirrhosis [55]. In line with this, our patients with MAFLD presented in advanced stages of liver disease (median CPS B8, median MELD 12) and predominately decompensated at baseline (65%). These patients had the highest percentage of HCC (15.0%) and death (45.%) relative to cohort size. Furthermore, previous studies also showed that MAFLD patients have an increased risk to develop PVT and we also found the highest ratio of PVT among patients with MAFLD in our cohort [56,57]. As the global prevalence of MAFLD is increasing, this population of patients with liver disease demands more attention [24,25,55]. Almost 4% of our patients presented with HCC at baseline, a rate unchanged in the last two decades despite medical advances [58,59]. None of these patients was eligible for curative treatment. Furthermore, only one of our 476 patients with cirrhosis was eligible for liver transplantation. This might be attributable to our center’s involvement in treating patients with CHC with (past and ongoing) intravenous substance use as well as patients with pathological alcohol intake, but also highlights the need to increase awareness of liver transplantation as well as linkage of patients to substance/alcohol use disorder programs.

In summary, our findings paint an alarming picture: The diagnosis of liver cirrhosis is frequently made at advanced stages with decompensation events commonly present. Patients with ALD are a special risk population of patients with cirrhosis, who are diagnosed at later stages of liver disease (higher CPS and MELD), have high-risk laboratory reports (low HDL, high CRP), regularly presented with varices or variceal bleeding, and have an unfavorable prognosis in general. Patients with viral hepatitis are diagnosed at earlier stages of cirrhosis and greatly benefit from antiviral treatment. Although a smaller subcohort in our study, the number of patients with cirrhosis due to MAFLD will be increasing in the future. We found that our patients with MAFLD presented at late stages and a high rate of liver disease-related complications like HCC and PVT. We argue that increased awareness of chronic liver disease among healthcare professionals, earlier diagnosis and consequentially, earlier treatment of liver disease may alleviate the burden of liver cirrhosis and its complications on healthcare systems worldwide.

## Supporting information

S1 File Supporting Information–contains all the supporting tables and figures. (DOCX)

S2 File (XLSX)
