# Supplementary material for: Late-stage presentation with decompensated cirrhosis is alarmingly common but successful etiologic therapy allows for favorable clinical outcomes
Source: PLoS One. 2023 Aug 24;18(8):e0290352. doi: 10.1371/journal.pone.0290352 (PMC10449133; doi:10.1371/journal.pone.0290352)
Supplement: S1 File — (DOCX) [file pone.0290352.s001.docx]

**Supplemental:**

|  | **Pts, %male** | **Age, years** | **BMI** | **Diabetes** | **FU (months)** | **death in FU** |
| --- | --- | --- | --- | --- | --- | --- |
| **overall** | 476 (72.5%) | 56.0 (45.5-67.0) | 25.7 (22.7-29.1) | 76 (16.0%) | 12.0 (3.0-25.0) | 116 (24.4%) |
| **ALD** | 211 (73.5%) | 58 (49.0-68.0) | 25.2 (21.9-28.5) | 31 (14.7%) | 9.0 (2.0-24.0) | 68 (32.2%) |
| **viral** | 163 (71.2%) | 48.0 (41.0-67.0) | 25.6 (22.6-28.4) | 20 (12.3%) | 14.0 (6.0-26.0) | 18 (11.0%) |
| **MAFLD** | 20 (65.0%) | 66.0 (56.3-74.5) | 31.0 (27.1-39.4) | 15 (75.0%) | 9.5 (0.8-39.0) | 9 (45.0%) |
| **AIH or cholestatic** | 10 (60.0%) | 72.5 (60.5-70.0) | 25.4 (22.4-31.0) | 1 (10.0%) | 24.5 (9.8-46.3) | 2 (20.0%) |
| **mixed** | 55 (81.8%) | 52.0 (43.0-59.3 | 26.0 (23.3-30.5) | 6 (10.9%) | 11.5 (5.3-18.3) | 14 (25.5%) |
| **other** | 17 (58.8%) | 76.0 (66.3-82.8) | 26.8 (23.5-28.4) | 3 (17.6%) | 8.0 (0.3-22.8) | 5 (29.4%) |

**Suppl. Table 1: Baseline characteristics of included patients with liver cirrhosis overall and by etiology.** ALD and viral cirrhosis were the biggest singular etiologies of cirrhosis. Most deaths overall occurred in the ALD cohort. However, relative to cohort size, highest rate of death was in the MAFLD cohort. As expected, BMI and diabetes rates were highest in the MAFLD population. Values given in absolutes (percent) and median (IQR). “Viral” includes chronic hepatitis B, C, and D, as well cirrhosis after clearance of viral hepatitis. “Other” includes cryptogenic cirrhosis, hemochromatosis, drug induced liver injury, and schistosomiasis.

|  | **overall** | **ALD** | **CHC SVR12** | **other** |
| --- | --- | --- | --- | --- |
| **CPS** | 7.1 vs 6.8 (p = 0.028) | 8.6 vs 7.6 (p < 0.001) | 5.8 vs 5.6 (p = 0.045) | 6.9 vs 7.0 (p = 0.507) |
| **MELD** | 12.8 vs 12.9 (p < 0.212) | 16.5 vs 15.8 (p = 0.186) | 9.1 vs 8.9 (p = 0.008) | 12.0 vs 12.8 (p = 0.873) |
| **Stiffness, kPa** | 29.4 vs 21.3 (p < 0.001) | n.e.d. | 26.2 vs 17.7 (p < 0.001) | 34.4 vs. 25.6 (p < 0.002) |
| **Platelet, G/L** | 143.8 vs 149.6 (p < 0.001) | 144.8 vs 143.7 (p = 0.47) | 146.6 vs 168.6 (p < 0.001) | 142.5 vs 137.8 (p = 0.965) |

**Suppl. Table 2:** **Comparison of the two main etiologies of cirrhosis in our cohort, patients with alcoholic liver disease or chronic hepatitis C undergoing treatment. Shown here are paired changes in Child-Pugh and MELD score, liver stiffness, and platelet count from baseline to last follow-up in paired measurements.** Overall, we found significant improvements in CPS, liver stiffness measured in transient elastography, and platelet count. Patients with alcohol-related liver disease (ALD) usually presented with higher CPS, but showed significant improvement over time. While patients with chronic hepatitis C, who achieved SVR12 (CHC SVR12) had lower CPS and MELD in general, but also showed significant improvements in both scores. Furthermore, liver stiffness and platelet count improved dramatically with treatment. “Other” included all other etiologies of liver cirrhosis besides ALD and CHC as well as overlaps between etiologies.

| **Characteristic** | **Number of patients** |
| --- | --- |
| Cirrhosis due to chronic hepatitis C (CHC) | 195 |
| - Viremic CHC (at baseline) | 179 (91.2% of 195) |
| - Cirrhosis after SVR12 (at baseline) | 7 (3.6%) |
| - No data on viremia | 9 (4.6%) |
| CHC genotype |  |
| - 1 | 95 (53.1 % of 179) |
| - 2 | 6 (3.4%) |
| - 3 | 54 (29.6%) |
| - 4 | 14 (7.8%) |
| - No data | 12 (6.7%) |
| CHC patients treated with DAAs^†^ | 125 |
| - SVR12 | 109 (87.2% of 125) |
| - Lost to follow-up | 12 (9.5%) |
| - Death | 3 (2.4%) |
| - On post-treatment surveillance | 1 (0.8%) |
| - Decompensated | 19 (17.4%) |
| Cirrhosis due to viral hepatitis | 218 |
| - CHC | 142 (65.1% of 218) |
| - CHB | 16 (7.3%) |
| - CHB + CHD | 4 (overlaps included in “CHB”) |
| - CHB + CHC | 4 (overlaps included in “CHB” and “CHC”) |
| - CHB + ALD | 7 (3.2%) |
| - CHC + ALD | 43 (19.7%) |
| - CHC + NAFLD | 5 (2.3%) |
| - CHC + schistosomiasis | 1 (0.5%) |

**Suppl. Table 3: cirrhosis due to viral hepatitis.** Our cohort included a total of 195 patients with cirrhosis due to hepatitis C, 179 of which were viremic at baseline, while seven had advanced chronic liver disease even after prior successful virus eradication. Predominant genotypes were genotype 1 and 54. We closely followed the course of 125 patients with cirrhosis due to chronic hepatitis C in absence of other causes. Nineteen patients had decompensated liver disease, when they received antiviral therapy, but still achieved SVR12. ^†^ Patients with additional etiologies of liver disease, e.g., ALD + viral hepatitis, also received antiviral treatment, if they were eligible and consent was obtained. However, these patients are not included in this analysis.

[placeholder for Suppl. Figure 1]

**Suppl. Figure 1: flow of dataset creation.** To create the cohort of cirrhotic patients, three automatic readouts were conducted for the observed timeframe: all laboratory reports for “ascitic fluid”, all endoscopy reports containing “varice/s”, and all transient elastographies with a liver stiffness ≥15kPa. After applying inclusion and exclusion criteria, a final cohort of 476 unique patients remained, half of them in a state of decompensated cirrhosis.
